# Differential expression of transfer RNA-derived small RNAs in IgA nephropathy

**DOI:** 10.1097/MD.0000000000023437

**Published:** 2020-11-25

**Authors:** Zhi-Feng Luo, Donge Tang, Hui-Xuan Xu, Liu-Sheng Lai, Jie-Jing Chen, Hua Lin, Qiang Yan, Xin-Zhou Zhang, Gang Wang, Yong Dai, Wei-Guo Sui

**Affiliations:** a The First School of Clinical Medicine, Southern Medical University, Guangzhou, Guangdong; b Department of Nephrology, Guangxi Key Laboratory of Metabolic Diseases Research, Affiliated No. 924 Military Hospital (Former No. 181 Military Hospital), Southern Medical University, Guilin, Guangxi; c Clinical Medical Research Center of The Second Clinical Medical College of Jinan University, Shenzhen People's Hospital, Shenzhen, Guangdong; d University of Chinese Academy of Sciences Shenzhen Hospital (Guangming), Shenzhen, Guangdong, China.

**Keywords:** bioinformatics analysis, high-throughput sequencing, iga nephropathy, peripheral blood mononuclear cells, quantitative real-time polymerase chain reaction, transfer RNA-derived small RNA

## Abstract

**Background:**

IgA nephropathy (IgAN) is one of the most common forms of primary glomerulonephritis. Recent studies have indicated that small noncoding RNAs, such as tRNA-derived small RNAs (tsRNAs), might be novel biomarkers for glomerulonephritis. We therefore investigated the potential roles and possible functions of the tsRNAs in IgAN.

**Method:**

Peripheral blood mononuclear cells (PBMCs) were extracted from blood samples of the patients with IgAN and healthy control groups. The expression profiles of tsRNAs were assessed by small RNA sequencing (RNA-Seq) in PBMCs of the IgAN and control groups. Dysregulated tsRNAs were selected for validation by quantitative real-time polymerase chain reaction (qRT-PCR). Target gene prediction and enrichment were performed by bioinformatics analysis.

**Results:**

The results revealed that 143 significantly upregulated and 202 significantly downregulated tsRNAs were differentially altered in the IgAN group compared with the control group. Five upregulated tsRNAs (tRF-Val-AAC-007, tRF-Ala-AGC-063, tRF-Gln-CTG-010, tRF-Tyr-GTA-011 and tRF-Thr-AGT-007) and 3 downregulated tsRNAs (tiRNA-Val-TAC-004, tRF-Gly-CCC-005 and tRF-His-GTG-006) were selected for validation by qRT-PCR; the results were consistent with the sequencing data. Gene Ontology (GO) analysis revealed that the target genes predicted by upregulated tsRNAs were mostly enriched in “nucleic acid metabolic process," “intracellular part," and “ion binding," whereas the target genes predicted by downregulated tsRNAs were mostly enriched in “regulation of cellular component organization," “membrane-bound organelle," and “ion binding." Kyoto Encyclopedia of Genes and Genomes pathway analysis revealed that the target genes predicted by upregulated tsRNAs were mostly enriched in “herpes simplex virus 1 infection," whereas the target genes predicted by downregulated tsRNAs were mostly enriched in “circadian rhythm

**Conclusions:**

The present study confirmed the differential expression of tsRNAs in patients with IgAN, and these dysregulated tsRNAs might be novel potential targets for the diagnosis and treatment of IgAN.

## 1 Introduction

IgA nephropathy (IgAN) is one of the most common forms of primary glomerulonephritis. The main pathological features of IgAN are IgA or IgA-based immune complexes deposited in the mesangial area, accompanied by membrane cell proliferation and matrix increase.^[1,2]^ Previous studies have shown that up to 40% of patients can progress to end-stage renal disease 5 to 25 years after diagnosis of IgAN.^[3,4]^ Thus, early diagnosis and treatment are critical for delaying or blocking the progression of IgAN. Histopathological diagnosis based on renal biopsy remains the only means of clinically validating IgAN and developing a reliable treatment plan. However, as an invasive procedure, renal biopsy, which has many contraindications and complications as well as poor reproducibility, is not conducive to the rapid diagnosis of disease and dynamic monitoring of therapeutic effects.^[5,6]^ Therefore, there is an urgent need for a noninvasive, sensitive, and specific biomarker to dynamically monitor the early diagnosis and treatment of IgAN.

Transfer RNA (tRNA) is an adaptor molecule that decodes mRNA and translates protein. Recent studies have demonstrated that tRNAs also serve as a major source of small noncoding RNAs with distinct and varied functions, such as tRNA-derived small RNAs (tsRNAs).^[7,8]^ tsRNAs can be broadly classified into two main groups: tRNA-related fragments (tRFs) generated from mature or precursor tRNA and tRNA halves (tiRNAs) generated by specific cleavage in the anticodon loops of mature tRNA, with characteristic sizes, nucleotide compositions, functions and biogenesis.^[9]^ tRFs and tiRNAs have been suggested to play important roles in gene regulation at the genome and chromosome level.^[9]^ A large number of studies have found that tRFs and tiRNAs are significantly increased in the presence of harmful stress such as starvation and oxidative stress in cells and can regulate the stability of messenger RNA (mRNA) by a mechanism similar to the mechanism of microRNA (miRNA) degradation of mRNA, thereby participating in important physiological processes such as the apoptosis escape, cell proliferation and DNA damage.^[10–12]^ Furthermore, recent research has shown that tRFs and tiRNAs are involved in the rat mesangial cell proliferation induced by transforming growth factor beta-1.^[13]^ These findings indicate that tsRNAs might provide potential novel diagnostic and therapeutic targets for IgAN.

In this study, we expect to observe the expression changes in tsRNAs in peripheral blood mononuclear cells (PBMCs) of patients with IgAN and then explore the role of tsRNAs in the pathogenesis of IgAN.

## 2 Materials and methods

### 2.1 Subjects

Seven patients who were diagnosed with IgAN by renal biopsy in The Shenzhen People's Hospital (Shenzhen, China) from January 2018 to August 2019 were included in this study. These patients had not received any systematic treatment before blood and urine sampling. Secondary IgAN caused by autoimmune diseases, infectious diseases, and cancer was excluded by renal biopsy and serological evidence. Other combined kidney diseases, such as diabetic nephropathy, obesity-related nephropathy, and qualitative nephritis, were also excluded. The histological lesions were classified according to Lee's classification system. In addition, 6 healthy volunteers whose age- and sex-matched the patients were recruited as a control group. Blood samples, urine samples, and clinical information of all subjects were collected before renal biopsy and then stored in a refrigerator at −80°C for later use.

This study is in line with the purpose of the Helsinki Declaration and was approved by the Medical Ethics Committees of the Shenzhen People's Hospital (Shenzhen, China) and the Guilin NO. 924 Military Hospital (Guilin, China). All study participants signed written informed consent.

### 2.2 PBMC extraction

PBMCs were isolated from blood samples of the IgAN and control group by density gradient centrifugation using Ficoll-Hypaque Solution (GE Healthcare, Marlborough, MA). After washing with phosphate buffered saline 3 times, PBMCs were resuspended in 2 mL of Roswell Park Memorial Institute 1640 medium containing 10% fetal bovine serum (Gibco, Grand Island, NY) and counted. PBMCs were identified by rapid Wright's staining. The cell concentration was adjusted to 1 × 10^6^ cells/mL, and then PBMCs were stored in a refrigerator at −80°C.

### 2.3 RNA isolation

Total RNA was extracted from PBMCs with TRIzol reagent (Invitrogen, Carlsbad, CA). The purity and concentration of total RNA samples were determined with a NanoDrop ND-1000 system (Thermo Fisher Scientific, Waltham, MA). The optical density (OD) 260/280 absorbance ratios of all the samples were between 1.8 and 2.0.

### 2.4 High-throughput sequencing

Total RNA samples were pretreated using the rtStar tRF & tiRNA Pretreatment Kit (Arraystar, Rockville, MD) to remove some RNA modifications that might interfere with small RNA-sequencing library construction. The total RNA of each sample was sequentially ligated to 3’ and 5’ small RNA adapters. Complementary DNA (cDNA) was then synthesized and amplified using Illumina's proprietary reverse transcription primers and amplification primers (Illumina, San Diego, CA).^[14]^ Subsequently, ∼134 to 160 bp polymerase chain reaction (PCR)-amplified fragments (corresponding to *∼*14–40 nt small RNAs) were extracted and purified from the polyacrylamide gel electrophoresis.^[15]^ The completed libraries were quantified by an Agilent 2100 Bioanalyzer using the Agilent DNA 1000 chip kit (Agilent Technologies, Santa Clara, CA). The libraries were denatured and diluted to a loading volume of 1.3 mL and loading concentration of 1.8 pmol/L. Finally, the diluted libraries were loaded onto a reagent cartridge and forwarded to sequencing run on an Illumina NextSeq 500 system using a NextSeq 500/550 V2 kit (Illumina, San Diego, CA), according to the manufacturer's instructions. Sequencing was carried out by running 50 cycles.

### 2.5 Raw sequencing data analysis

Raw sequencing data generated from Illumina NextSeq 500 system that pass the Illumina chastity filter are used for the following analysis.^[14]^ After Illumina quality control, the sequencing reads were 5’, 3’-adaptor trimmed, and reads were discarded (length *<*14 nt or length *>*40 nt) with Cutadapt software (https://cutadapt.readthedocs.io). The trimmed reads were then aligned to the mature-tRNA and pre-tRNA sequences from GtRNAdb (http://gtrnadb.ucsc.edu/) using Bowtie software (http://bowtie-bio.sourceforge.net). The exactly matched reads were thought to be tsRNAs. The abundance of tsRNAs is evaluated using their sequencing counts and is normalized as counts per million (CPM) of total aligned reads. tsRNA expression profiling and differential expression analysis were calculated by the average CPM. Fold change (FC = 2^log2(IgANCPM^^–^^Control CPM)^) was used for screening differentially expressed tsRNAs. FC >1.5 was considered significantly different expression, and these tRNAs were selected for further analysis.

### 2.6 Quantitative real-time PCR

To validate the tsRNA sequencing results, the expression of selected tsRNAs was examined by quantitative real-time polymerase chain reaction (qRT-PCR). Total RNA isolated from PBMCs was pretreated with the rtStar tRF&tiRNA Pretreatment Kit (Arraystar, Rockville, MD), and the cDNA was synthesized using the rtStar First-Strand cDNA Synthesis Kit (3’ and 5’ adaptor; Arraystar, Rockville, MD) according to the manufacturer's protocol. The primer pairs of each gene are listed in Table 1 (KangChen Bio-tech, Shanghai, China). qRT-PCR was performed using a ViiA 7 Real-time PCR System (Applied Biosystems, Foster City, CA) with 2 × PCR Master Mix (Arraystar, Rockville, MD); thermal cycling conditions were 95°C for 10 minutes, followed by 40 cycles of 95°C for 10 seconds and 60°C for 60 seconds. The relative expression level of tsRNAs was calculated using the comparative Ct (2^−ΔΔCt^) method and normalized to U6.

**Table 1:** Sequence of the primers for candidate tsRNAs and internal control.

Gene name	Primer sequence	Annealing temperature (°C)	Product length, bp
tRF-Val-AAC-007	F: 5’ GATCGTTTCCGTAGTGTAGTGG 3’	60	43
	R: 5’ TGTGCTCTTCCGATCTGATG 3’		
tRF-Ala-AGC-063	F: 5’ GACGATCTCGATCCCCAGTA 3’	60	44
	R: 5’ GTGCTCTTCCGATCTTGGTG 3’		
tRF-Gln-CTG-010	F: 5’ ACAGTCCGACGATCCAAATCTC 3’	60	48
	R: 5’ GCTCTTCCGATCTTGGAGGTT 3’		
tRF-Thr-AGT-007	F: 5’ AGTCCGACGATCATCCCAGC 3’	60	41
	R: 5’ TCTTCCGATCTCGGAGGCAC 3’		
tRF-Tyr-GTA-011	F: 5’ CTACAGTCCGACGATCATCCG 3’	60	47
	R: 5’ GCTCTTCCGATCTTGGTCCTC 3’		
tiRNA-Val-TAC-004	F: 5’ GATCGGTTCCATAGTGTAGTGG 3’	60	45
	R: 5’ CCGATCTAAAGCAGACGTGATA 3’		
tRF-Gly-CCC-005	F: 5’ GATCGCATTGGTGGTTCAAT 3’	60	47
	R: 5’ TCTTCCGATCTAGGCGAGAAT 3’		
tRF-His-GTG-006	F: 5’ GATCGCCGTGATCGTATAGTG 3’	60	51
	R: 5’ CGTGTGCTCTTCCGATCTCA 3’		
U6	F: 5’ GCTTCGGCAGCACATATACTAAAAT 3’	60	89
	R: 5’ CGCTTCACGAATTTGCGTGTCAT 3’		

### 2.7 Target gene prediction, Gene Ontology and Kyoto Encyclopedia of Genes and Genomes pathway analyses

The target genes of tsRNAs were predicted by miRanda (http://www.microrna.org) and TargetScan (http://www.targetscan.org), and only genes predicted by both software were considered targets of tsRNAs. Gene Ontology (GO) (http://www.geneontology.org/) was used to describe the attributes of differentially expressed genes. Pathway analysis was applied to identify significantly enriched differentially expressed gene pathways on the basis of the Kyoto Encyclopedia of Genes and Genomes (KEGG) database (http://www.genome.jp/kegg/). Fisher exact test was performed to analyze the overlap between the GO/KEGG annotation list and the differentially expressed genes list. *P* < .05 was applied to denote the significant enrichment of GO terms/pathways.

### 2.8 Statistical analysis

For continuous variables, data with a normal distribution are presented as the mean ± SD, and the differences between the 2 groups were evaluated by an independent-samples *t* test. The difference in the sex distribution between the 2 groups was evaluated using the χ^2^ test. All statistical analyses were performed with SPSS version 25.0 (IBM Corp., Chicago, IL). Differences yielding *P* < .05 were considered statistically significant.

## 3 Results

### 3.1 Baseline characteristics of study participants

The clinical and demographic characteristics of patients with IgAN and healthy controls in this study are summarized in Table 2. Compared with the controls, the levels of urinary red blood cells and proteinuria were significantly increased (all *P* < .05). There were no significant differences in the average age, sex distribution, serum creatinine, blood urea nitrogen, serum IgA, serum C3, and serum C4 between patients and controls (all *P* > .05). According to Lee's glomerular grading system, all patients were above grade III.

**Table 2:** Clinical baseline data of patients with IgAN and healthy controls.

Characters	IgAN (*n *= 7)	Controls (*n *= 6)	*P*
Age, y	30.14 ± 10.70	27.50 ± 10.43	.662
Sex (male/female)	2/5	2/4	.853
URBC (/HPF)	255.71 ± 24.37	3.00 ± 1.41	.000
Proteinuria, g/day	1.35 ± 0.52	0.24 ± 0.12	.000
SCr, μmol/L	93.72 ± 18.02	91.49 ± 9.67	.792
BUN, mmol/L	9.45 ± 1.36	9.18 ± 0.59	.664
Serum IgA, g/L	2.17 ± 1.10	1.98 ± 0.75	.724
Serum C3, g/L	1.22 ± 0.12	1.33 ± 0.24	.296
Serum C4, g/L	0.40 ± 0.08	0.39 ± 0.13	.889
Lee's classification	≥ III		

### 3.2 Catalog of tsRNAs expressed in patients with IgAN and healthy controls

**Figure 1:**
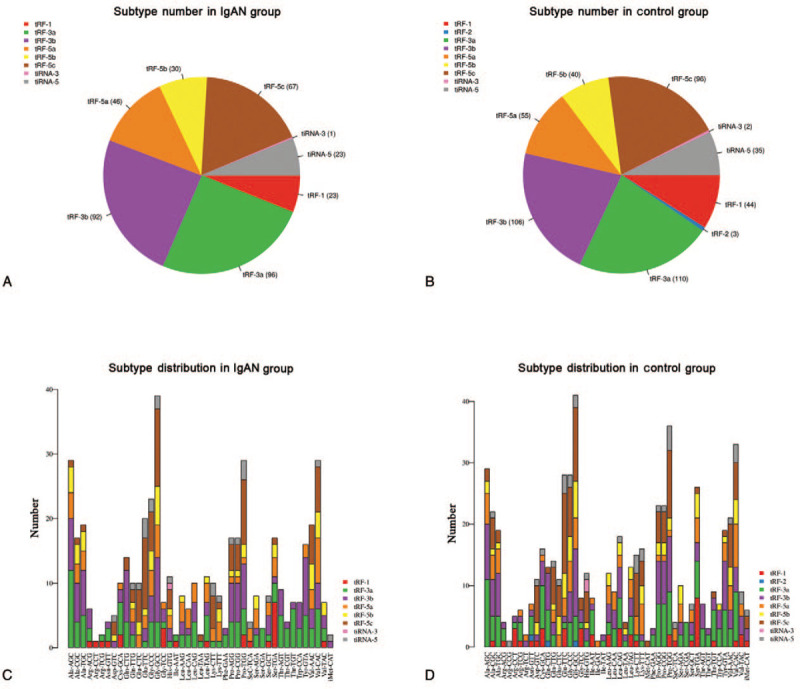
Transfer RNA-derived small RNAs (tsRNA) subtype distribution between the IgAN group and control group by small RNA-Seq. Pie chart of the distribution of the number of tsRNA subtypes in the IgAN (A) and control (B) groups. tsRNA subtype distribution in the IgAN group (C) and control group (D). The *x* axes represent tRNA isodecoders, and the Y axes show the number of all tsRNA subtypes against tRNA isodecoders in the stacked plots. IgAN = IgA nephropathy.

The subtype numbers of tsRNA transcripts in both the IgAN and control groups were estimated to investigate whether tsRNA production was altered in PBMCs of patients with IgAN. The pie chart shows the distribution of the number for each subtype of tsRNA in which the average CPM of the group is not <20. In patients with IgAN, 23 tRF-1, 96 tRF-3a, 92 tRF-3b, 46 tRF-5a, 30 tRF-5b, 67 tRF-5c, 1 tiRNA-3, and 23 tiRNA-5 were identified (Fig. 1A). In the control group, 44tRF-1, 3 tRF-2, 110 tRF-3a, 106 tRF-3b, 55 tRF-5a, 40 tRF-5b, 96 tRF-5c, 2 tiRNA-3, and 35 tiRNA-5 were identified (Fig. 1B). In addition, the numbers of tsRNAs derived from the variable anticodon tRNAs are shown in the stacked plots (Fig. 1C, D).

### 3.3 Differential expression of tsRNAs in patients with IgAN and healthy controls

**Figure 2:**
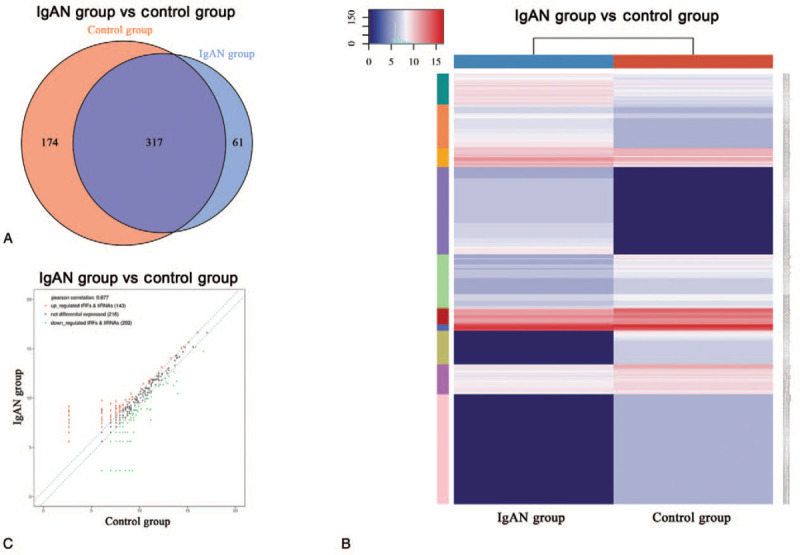
Differentially expressed transfer RNA-derived small RNAs (tsRNAs) between the IgAN group and the control group. Venn diagram shows the number of commonly expressed and specifically expressed tsRNAs in the IgAN group and control group (A). The commonly expressed tsRNAs represent the average counts per million (CPM) values, which were not <20 in both groups, and the specifically expressed tsRNAs represent the CPM values, which were >20 in one group and <20 in the other group. Heat map and hierarchical clustering analysis of differentially expressed tsRNAs between the IgAN group and control group (B). The color in the panel represents the relative expression level (log2-transformed). The color scale is shown below: blue represents an expression level below the mean, and red represents an expression level above the mean. The scatter plots of differentially expressed tsRNAs (C). The values of the *x* and *y* axes in the scatter plot are the averaged CPM values of each group (log2 scaled). tsRNAs above the top line (red dots, upregulation) or below the bottom line (green dots, downregulation) indicate >1.5-fold change between the 2 compared groups. Gray dots indicate nondifferentially expressed tsRNAs. IgAN = IgA nephropathy.

The Venn diagram shows the numbers of all expressed tsRNAs in the 2 groups in a previous study. In total, 317 commonly expressed tsRNAs were identified in the patients with IgAN and the control group, whereas 61 and 174 specifically expressed tsRNAs were identified in the patients with IgAN and the control group, respectively (Fig. 2A). To analyze the different expression levels of tsRNAs among patients with IgAN and healthy controls, the threshold of FC >1.5 was used for the RNA-Seq readout, and all differentially expressed tsRNAs are shown in the heat map (Fig. 2B). Overall, 143 significantly upregulated and 202 significantly downregulated tsRNAs were differentially altered in the IgAN group compared with the control group. The scatter plot demonstrates that the expression of the 2 groups correlated well between the 2 samples (Fig. 2C). In particular, tRF-Val-AAC-007 and tRF-His-GTG-009 are the most significant up- and downregulated tsRNAs in patients with IgAN based on their fold changes (Table 3).

**Table 3:** Top 10 upregulated and downregulated tsRNAs in patients with IgAN.

tsRNA_ID	Sequence	Type	Length, bp	Regulation	Fold change
tRF-Val-AAC-007	GTTTCCGTAGTGTAGTGGTCATC	tRF-5b	23	Up	89.512
tRF-Ala-AGC-063	TCGATCCCCAGTACCTCCACCA	tRF-3b	22	Up	69.086
tRF-Gln-CTG-010	CAAATCTCGGTGGAACCTCCA	tRF-3b	21	Up	69.086
tRF-Tyr-GTA-011	ATCCGGCTCGGAGGACCA	tRF-3a	18	Up	69.086
tRF-Ala-AGC-039	GGGGATGTAGCTCAGTGGTAGAG	tRF-5b	23	Up	62.277
tRF-Thr-AGT-007	ATCCCAGCGGTGCCTCCG	tRF-3a	18	Up	48.660
tRF-Ala-AGC-038	GGGGATGTAGCTCAGTGGTAGA	tRF-5b	22	Up	35.043
tRF-Cys-GCA-046	TCCAGGTGCCCCCTCCA	tRF-3a	17	Up	35.043
tRF-Gly-GC-031	GCATGGGTGGTTCAG	tRF-5a	15	Up	35.043
tRF-Ala-AGC-059	TCCCCAGCACCTCCACCA	tRF-3a	18	Up	28.234
tRF-His-GTG-009	GCCGTGATCGTATAGTGGTTAGTACTCTGCGT	tRF-5c	32	Down	0.010
tiRNA-Val-TAC-004	GGTTCCATAGTGTAGTGGTTATCACGTCTGCTTT	tiRNA-5	34	Down	0.013
tRF-Gly-CCC-005	GCATTGGTGGTTCAATGGTAGAATTCTCGCCT	tRF-5c	32	Down	0.017
tRF-His-GTG-006	GCCGTGATCGTATAGTGGTTAGTACTCTG	tRF-5c	29	Down	0.017
tRF-Ser-TGA-013	AACCCTGCTCGCTGCGCCA	tRF-3b	19	Down	0.025
tRF-Val-TAC-005	CAGAGTGTAGCTTAAC	tRF-5a	16	Down	0.025
tRF-Gln-TTG-011	TAGGATGGGGTGTGATAGGTGGCACGGAGAA	tRF-5c	31	Down	0.033
tRF-Lys-TTT-011	GCCCGGATAGCTCAGTCGGTAGAGCATCAGAC	tRF-5c	32	Down	0.033
tiRNA-Lys-TTT-001	CACTGTAAAGCTAACTTAGCATTAACCTT	tiRNA-5	29	Down	0.049
tiRNA-Val-CAC-002	GTTTCCGTAGTGTAGCGGTTATCACATTCGCCTC	tiRNA-5	34	Down	0.049

### 3.4 Validation of the sequencing data of tsRNAs in patients with IgAN and healthy controls by qRT-PCR

**Figure 3:**
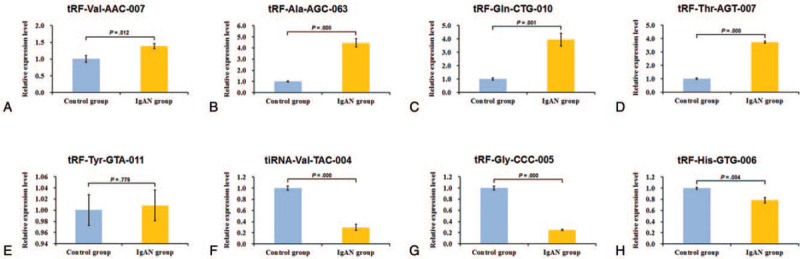
The relative expression levels of selected tsRNAs validated by quantitative real-time polymerase chain reaction (qRT-PCR) in the IgA nephropathy (IgAN) group and control group. The qRT-PCR results for selected tsRNA expression were analyzed by a comparative Ct (2^−ΔΔCt^) method and are shown as relative levels. tRF-Val-AAC-007 (A), tRF-Ala-AGC-063 (B), tRF-Gln-CTG-010 (C), tRF-Thr-AGT-007 (D), tiRNA-Val-TAC-004 (F), tRF-Gly-CCC-005 (G), and tRF-His-GTG-006 (H) were significantly different (all *P* < .05) between the IgAN group and the control group. The expression of tRF-Tyr-GTA-011 (E) showed an increasing trend, which was similar to the sequencing results, while the alterations were not statistically significant (*P* = .779). Means ± SD. *P* < .05 indicates statistical significance. tiRNA = tRNA halve, tRF = tRNA-related fragment, tsRNA = transfer RNA-derived small RNAs.

To validate the tsRNA-Seq results, 5 upregulated tsRNAs (tRF-Val-AAC-007, tRF-Ala-AGC-063, tRF-Gln-CTG-010, tRF-Tyr-GTA-011, and tRF-Thr-AGT-007) and 3 downregulated tsRNAs (tiRNA-Val-TAC-004, tRF-Gly-CCC-005, and tRF-His-GTG-006) were selected and confirmed by qRT-PCR. Compared with the control group, the results demonstrated that the expression levels of tRF-Val-AAC-007, tRF-Ala-AGC-063, tRF-Gln-CTG-010, and tRF-Thr-AGT-007 were significantly upregulated (Fig. 3A–D), whereas the expression levels of tiRNA-Val-TAC-004, tRF-Gly-CCC-005, and tRF-His-GTG-006 were significantly downregulated in the IgAN group (Fig. 3F–H) (all *P* < .05). Although the expression trend of tRF-Tyr-GTA-011 was similar to the tsRNA-Seq results, there was no significant difference for tRF-Tyr-GTA-011 between the 2 groups due to the small sample size (Fig. 3E) (*P* = .779).

### 3.5 Prediction of target genes for selected tsRNAs and functional differencially expressed tsRNA enrichment analysis

To explore the potential functions and mechanism of these dysregulated tsRNAs in the PBMCs of patients with IgAN and healthy controls, first, 2 types of algorithms named TargetScan and miRanda were applied to predict tsRNA targets. tRF-Val-AAC-007 was predicted to have 854 target genes; tRF-Ala-AGC-063 was predicted to have 258 target genes; tRF-Gln-CTG-010 was predicted to have 422 target genes; tRF-Thr-AGT-007 was predicted to have 2714 target genes; tRF-Tyr-GTA-011 was predicted to have 573 target genes; tiRNA-Val-TAC-004 was predicted to have 1738 target genes; tRF-Gly-CCC-005 was predicted to have 1566 target genes; and tRF-His-GTG-006 was predicted to have 528 target genes.

**Figure 4:**
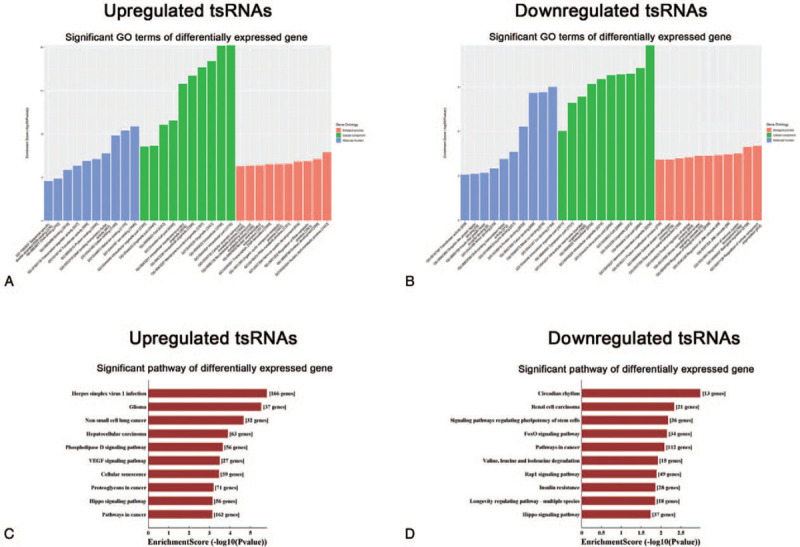
GO analysis (A, B) and KEGG pathway analysis (C, D) of target genes based on selected tsRNAs. The bar graph shows the top ten enrichment score values for the significant enrichment terms or pathway. tsRNAs = transfer RNA-derived small RNAs, GO = Gene Ontology, VEGF = vascular endothelial growth factor.

Furthermore, GO and KEGG pathway analyses were performed based on the predicted target genes. The GO analysis revealed that the target genes of upregulated tsRNAs were mostly enriched in “nucleic acid metabolic process" (biological process, GO: 0090304), “intracellular part" (cellular component, GO: 0044424) and “ion binding" (molecular function, GO: 0043167) (Fig. 4A), whereas the target genes of downregulated tsRNAs were mostly enriched in “regulation of cellular component organization" (biological process, GO: 0051128), “membrane-bound organelle" (cellular component, GO:0043227), and “ion binding" (molecular function, GO: 0043167) (Fig. 4B). KEGG pathway analysis revealed that the target genes of upregulated tsRNAs were mostly enriched in “herpes simplex virus 1 infection" (Fig. 4C), whereas the target genes of downregulated tsRNAs were mostly enriched in “circadian rhythm" (Fig. 4D).

## 4 Discussion

Due to the maturity and wide application of molecular cell biology, gene chip, protein profiling, metabolomics, and other technologies, numerous current studies found that interleukins, podocyte components, immune complexes, complement factors, and miRNAs in peripheral blood or urine of patients with IgAN were closely related to the development of IgAN and were indicated to be potential biomarkers for the diagnosis and evaluation of IgAN.^[16–20]^ However, there are no definite biomarkers for routine clinical testing. With technological advances in sequence technology, a large number of recent studies have found that tsRNAs (tRF and tiRNA) derived from tRNA small fragments involve various cellular processes, such as proliferation, apoptosis, protein synthesis control, and RNA interference, and are involved in the physiological processes of cancer, neurodegenerative diseases, and viral infections.^[21–23]^ Increasing evidence shows that tsRNAs may constitute potential novel molecular targets that regulate pathological processes.^[24]^

To explore whether tsRNAs play a potential role in IgAN, the expression profiling of tsRNAs in the PBMCs of patients with IgAN and healthy controls was detected by high-throughput sequencing in our present study. Based on sequencing results, 354 tRFs and 24 tiRNAs were detected in the PBMCs of IgAN, whereas 454 tRFs and 37 tiRNAs were detected in the PBMCs of healthy controls. Then, 143 significantly upregulated and 202 significantly downregulated tsRNAs were screened from cell samples of the 2 groups. Successively, of these, 5 upregulated tsRNAs and 3 downregulated tsRNAs were selected for further validation by qRT-PCR, and the results demonstrated that tRF-Val-AAC-007, tRF-Ala-AGC-063, tRF-Gln-CTG-010, and tRF-Thr-AGT-007 were significantly upregulated in the PBMCs of IgA patients compared with healthy controls, and tRF-Tyr-GTA-011 was also upregulated, although not significantly. In addition, tiRNA-Val-TAC-004, tRF-Gly-CCC-005, and tRF-His-GTG-006 were significantly downregulated in the PBMCs of IgA patients compared with healthy controls. These results were consistent with the sequencing data. However, to the best of our knowledge, the potential role of tsRNAs in IgAN has not been reported.

Although dysregulated tsRNAs were found in the PBMCs of patients with IgAN based on sequencing data in this study, whether these 8 dysregulated tsRNAs (tRF-Val-AAC-007, tRF-Ala-AGC-063, tRF-Gln-CTG-010, tRF-Thr-AGT-007, tRF-Tyr-GTA-011, tiRNA-Val-TAC-004, tRF-Gly-CCC-005, and tRF-His-GTG-006) are involved in the development and/or progression of human IgAN remains unknown and needs to be further investigated in large clinical studies. Therefore, the results of this study are based only on bioinformatics analysis. GO analysis showed that “ion binding" is the most enriched molecular function of the target genes predicted by validated upregulated and downregulated tsRNAs. A previous study demonstrated that anionic charge plays an important role in the deposition of IgA1 onto mesangial cells, and the binding of IgA to human mesangial cells is charge-dependent.^[25]^ These results indicated that 8 validated tsRNAs might play functional roles in the pathogenesis of IgAN by binding. In addition, “nucleic acid metabolic process" and “regulation of cellular component organization" are the most enriched biological processes of those upregulated tsRNAs and downregulated tsRNAs, respectively, whereas previous studies found that tsRNAs could regulate the stability of mRNA and were associated with mesangial cell proliferation,^[12,13]^ suggesting that 8 validated tsRNAs might be involved in the pathological process of IgAN. On However, among the top 10 enrichment pathways of KEGG analysis based on the target genes predicted by validated dysregulated tsRNAs in the present study, “herpes simplex virus 1 infection,"^[26]^ “VEGF signaling pathway,"^[27]^ “cellular senescence,"^[28]^ “hippo signaling pathway,"^[29]^ “circadian rhythm,"^[30]^ “FoxO signaling pathway,"^[31]^ and “insulin resistance"^[32]^ have been reported to be involved in the pathogenesis of IgAN. This indicated that tRF-Val-AAC-007, tRF-Ala-AGC-063, tRF-Gln-CTG-010, tRF-Thr-AGT-007, tRF-Tyr-GTA-011, tiRNA-Val-TAC-004, tRF-Gly-CCC-005, and tRF-His-GTG-006 could potentially be used as diagnostic and therapeutic biomarkers for IgAN. However, further experiments are required to confirm these results.

It should be noted that there are some limitations to our present study. First, although the aberrant expression of tRF-Val-AAC-007, tRF-Ala-AGC-063, tRF-Gln-CTG-010, tRF-Thr-AGT-007, tRF-Tyr-GTA-011, tiRNA-Val-TAC-004, tRF-Gly-CCC-005, and tRF-His-GTG-006 in the PBMCs of IgAN has been confirmed by sequencing and qRT-PCR, their expression and roles in the pathogenesis of IgAN have not been systematically demonstrated by functional experiments. Second, because the number of cases was small and only IgAN grade III or higher samples were included in this study, the changes in altered tsRNAs at different time points, especially at different stages of IgAN onset, could not be observed. In addition, although we have confirmed the abnormal expression of these 8 tsRNAs in IgAN, it is not yet known whether there are abnormal expressions in other primary glomerulonephritis such as idiopathic membranous nephropathy, minimal change nephropathy, and focal segmental glomurular sclerosis. Therefore, the relationship between tsRNAs and other glomerulonephritis needs to be further explored in the future.

In summary, our present study showed the expression profiles of altered tsRNAs in the PBMCs of patients with IgAN through high-throughput sequencing analysis and performed bioinformatics analysis, initially revealing the potential role of altered tsRNAs in the pathogenesis of IgAN. Although precise functional investigations should be explored in the future, our present study could provide a theoretical basis for further exploration of the biological functions of these novel tsRNAs in the pathogenesis of IgAN.

## Acknowledgments

The authors thank the family and the patients for their cooperation.

## Author contributions

**Conceptualization:** Zhi-Feng Luo, Donge Tang, Yong Dai, Wei-Guo Sui.

**Data curation:** Zhi-Feng Luo, Donge Tang, Hui-Xuan Xu.

**Funding acquisition:** Liu-Sheng Lai, Yong Dai, Wei-Guo Sui.

**Investigation:** Zhi-Feng Luo, Hui-Xuan Xu.

**Methodology:** Zhi-Feng Luo, Donge Tang.

**Resources:** Jie-Jing Chen, Qiang Yan, Xin-Zhou Zhang, Gang Wang.

**Software:** Zhi-Feng Luo

**Validation:** Hua Lin

**Writing – original draft:** Zhi-Feng Luo, Donge Tang

**Writing – review & editing:** Zhi-Feng Luo, Donge Tang, Yong Dai, Wei-Guo Sui.
